# Study protocol of an international patient-led registry in patients with pulmonary fibrosis using online home monitoring: I-FILE

**DOI:** 10.1186/s12890-023-02336-4

**Published:** 2023-02-02

**Authors:** Gizal Nakshbandi, Catharina C. Moor, Katerina Antoniou, Vincent Cottin, Anna-Maria Hoffmann-Vold, Edwin A. Koemans, Michael Kreuter, Philip L. Molyneaux, Wim A. Wuyts, Marlies S. Wijsenbeek

**Affiliations:** 1 Centre of Excellence for Interstitial Lung Diseases and Sarcoidosis, Department of Respiratory Medicine, Erasmus University Medical Centre, Dr. Molewaterplein 40, 3015 GD Rotterdam, The Netherlands; 2 Dept of Thoracic Medicine and Laboratory of Cellular and Molecular Pneumonology, Medical School, University of Crete, Crete, Greece; 3 Department of Respiratory Medicine, National Coordinating Reference Center for Rare Pulmonary Diseases, Louis Pradel Hospital, Lyon, France; 4 UMR 754, Claude Bernard University, Lyon, France; 5 Department of Rheumatology, Oslo University Hospital, Oslo, Norway; 6 Elshout, The Netherlands; 7 Center for Interstitial and Rare Lung Diseases, Thoraxklinik, University of Heidelberg, Heidelberg, Germany; 8 German Center for Lung Research, Heidelberg, Germany; 9 Department of Pneumology, RKH Clinics Ludwigsburg, Ludwigsburg, Germany; 10 Interstitial Lung Disease Unit, Royal Brompton and Harefield Hospitals, Guy’s and St Thomas’ NHS Foundation Trust, London, UK; 11 Unit for Interstitial Lung Diseases, Department of Respiratory Diseases, University Hospitals Leuven, Leuven, Belgium

**Keywords:** eHealth, Home monitoring, Pulmonary fibrosis, Spirometry, Health related quality of life, Interstitial lung diseases

## Abstract

**Background:**

Pulmonary fibrosis (PF) is caused by a heterogeneous group of diseases, with a high inter-individual variability in disease trajectory. Identifying disease progression in patients with PF has impact on clinical management decisions. However, strategies to early identify and predict disease progression for these patients are currently lacking. In this study, we aim to assess long-term FVC change in patients with PF measured with home spirometry, and evaluate the feasibility of a multinational patient-led registry in PF. In addition, we will assess validity of patient-reported outcomes (PROMs) for the different subgroups of patients with PF.

**Methods:**

In this international, prospective, multicenter, observational study, we aim to include 700 patients across seven European countries. Patients will monitor their disease course for a period of two years using an online home monitoring program (I-FILE), which includes home spirometry, pulse oximetry, and PROMs. Results will be directly sent to the hospital via the online application. Patients will be asked to perform daily home spirometry and pulse oximetry in the first three months, followed by once weekly measurements for a period of two years. PROMs will be completed in the online I-FILE application every six months, including the King’s brief Interstitial Lung Disease Health Status, The EuroQol five dimensions five-level, Visual Analogue Scales on cough, dyspnea, fatigue and general complaints, Leicester Cough Questionnaire, Fatigue Assessment Scale, Work Productivity and Activity Impairment Questionnaire, Global Rating of Change Scale, and Living with Pulmonary Fibrosis questionnaire.

**Discussion:**

This study will provide much needed insights in disease trajectories of the different subgroups of patients with PF. Simultaneously, the I-FILE study will yield valuable information on the use and feasibility of home-based data collection. This international patient-led registry will facilitate trans-border collaboration to further optimize care and research for patients with PF.

*Trial registration:* The study was registered on the 12th of March 2020 in the International Clinical Trial Registry, www.clinicaltrials.gov; Identifier: NCT04304898.

**Supplementary Information:**

The online version contains supplementary material available at 10.1186/s12890-023-02336-4.

## Background

Pulmonary fibrosis (PF) is a manifestation of different interstitial lung diseases, characterized by fibrotic remodeling of the lung, leading to pulmonary function loss [1, 2]. Disease course and prognosis of PF widely vary between individuals and due to different underlying etiologies [3–6]. Symptoms such as cough, dyspnea, impaired exercise tolerance, fatigue, anxiety and depression, significantly impair health-related quality of life (HRQOL) of patients with PF [7–9]. With appropriate and timely management, some forms of PF have the potential for stabilization or, in a subgroup with more inflammatory disease, even improvement [1]. However, a subset of patients have a progressive disease course, characterized by increasing symptoms, decline in pulmonary function and HRQOL, and ultimately early mortality [2–6, 10, 11]. With a median survival of two to three years without treatment, idiopathic pulmonary fibrosis (IPF) is the most progressive form of PF [12]. Nevertheless a group of patients with other forms of pulmonary fibrosis have a similar rapidly progressive disease course. New guidelines have introduced the term progressive pulmonary fibrosis (PPF) for this progressive phenotype of PF in patients with a diagnosis other than IPF. Due to the heterogeneity of PF, predicting the disease course for individual patients remains challenging. Especially for the more rare and ultra-rare forms of PF, detailed data about disease behavior and trajectory is currently lacking. Therefore, more comprehensive data for the different forms of PF is needed.

Nowadays, identifying disease progression has direct treatment implications. Anti-fibrotic medication is the mainstay of treatment for IPF, as it reduces pulmonary function decline and prolongs survival [13–15]. More recently, studies demonstrated beneficial effects of the anti-fibrotic medications Nintedanib and Pirfenidone for PPF [16–19]. This has led to the approval of anti-fibrotic treatment (Nintedanib) for this patient group [16, 17]. However, data on timing of treatment initiation, and combination with immunomodulatory treatment in patients with PPF are still scarce. Moreover, real-life data about adverse events and effectiveness in patients with comorbidities or severely impaired pulmonary function, and not eligible for clinical trials, are currently also not available.

In current guidelines, disease progression is defined by a combination of worsening symptoms, radiological and/or physiological progression [20]. In-hospital forced vital capacity (FVC) trends are accepted as current standard to monitor physiological disease progression. Nevertheless, FVC measurements have an inherent variability and are usually measured at low frequency during visits to the pulmonologist, possibly delaying detection of disease progression, and consequently, management decisions. Online home monitoring, using eHealth technology, enables more frequent measurements of pulmonary function and symptoms by patients at home, which will help to get a more detailed overview of disease course in individual patients. Home monitoring in PF has gained increasing interest in recent years, with studies demonstrating its feasibility and reliability in this patient population, despite technical and analytical issues [18, 21–25]. Results of an international survey revealed that ILD healthcare providers are positive towards the use of online home monitoring for daily care, but also for research and registry purposes [26]. A large online multinational patient registry could be a major step towards the better understanding of PF heterogeneity, and thus improve its management. eHealth technology can help lift the burden of frequent data collection, and provide a more practical way of data collection. It could facilitate trans-border collaboration and pooling of data in a patient registry, as distances are bridged online.

In the I-FILE study, we aim to gain more insights in disease behavior and response to treatment in patients with PF using online home monitoring. In addition, we will evaluate the feasibility of a patient-led registry in PF.

## Methods

### Objectives

The main aim of this study is to assess long-term FVC change in patients with PF measured with home spirometry. Our secondary aims are to evaluate feasibility, adherence, patient and healthcare provider experiences and satisfaction, and to assess validity existing PROMs in patients with PF.

### Study design and participants

The I-FILE study is an ongoing prospective multicenter, multinational, observational pilot study (NCT04304898). Patients with ILD, newly diagnosed in a multidisciplinary discussion (MDD), and PF on high-resolution computed tomography (HRCT), will be included in both community and tertiary centers in Belgium, France, Germany, Greece, the Netherlands, Norway and The United Kingdom. The trial is currently underway, and patients are being recruited. We aim to include approximately 700 patients in total. Patients will be considered newly diagnosed, when the diagnosis has been established within six months before inclusion in an MDD. In addition, patients should be either treatment naïve or have received treatment for their PF for a maximum of one month before inclusion. For more detail, inclusion—and exclusion criteria are shown in Table 1.

**Table 1:** Inclusion and exclusion criteria of the I-FILE study

Inclusion criteria	Exclusion criteria
Newly diagnosed patients with ILD and PF on HRCT Pulmonary fibrosis present on HRCT according to current guidelines, confirmed by a thoracic radiologist at the MDD [20] MDD diagnosis ≤ 6 months before inclusion Treatment for ILD ≤ than 1 month	Not able to speak, read or write in the native language of the country where the patient is included
	Not able to comply to the study protocol, according to the judgement of the investigator and/or patient
	No access to the internet

*ILD* Interstitial lung disease*, PF* Pulmonary fibrosis*, MDD* Multi-disciplinary discussion*, HRCT* High-resolution computed tomography

### Study procedures

In the I-FILE study, patients will use an online home monitoring program, consisting of the online I-FILE application integrated with home spirometry, for two years. If eligible for participation, patients will receive information on the I-FILE study by their treating physician. Written informed consent will be obtained before start of the study. At time of inclusion, patients will be instructed on the use of the I-FILE application and spirometer. Patients will perform once daily home spirometry and oximetry measurements for the first three months, followed by once weekly measurements (three consecutive home spirometry and one oximetry measurement). In addition, patients are asked to complete patient reported outcomes (PROMs) in the online application every six months. Permission will be asked separately to reassess medical records and online data for another five years after the end of the study. After two years, patients will be asked to fill in a twelve-item survey on their experiences with the home monitoring program. After two years, patients are offered the possibility to continue using the system if they wish.

**Fig. 1:**
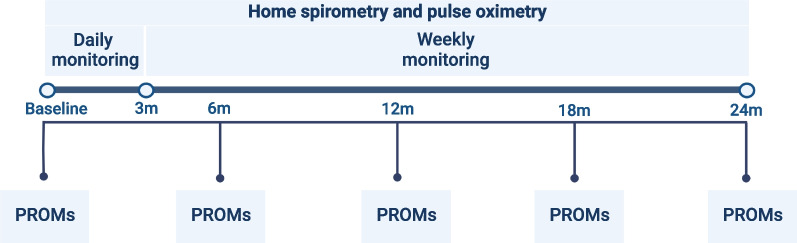
Study Flow I-FILE study. Home monitoring of patients with progressive pulmonary fibrosis using home spirometry, pulse oximetry, and patient reported outcome measures (PROMs)

Baseline characteristics, such as patient demographics, clinical characteristics, medication, data on HRCT, histology, comorbidities, hospital-based pulmonary function tests, and laboratory results will be collected at time of inclusion. An overview of the data collection is shown in Additional file 1: Table S1. No adjustments will be made in regular care of participating patients. Every six months, during outpatient clinic visits, patients will be asked about hospitalizations, changes in medication use and side-effects of medication for PF. Hospital-based spirometry, consisting of FVC, Forced Expiratory Volume in 1 s (FEV1), and diffusion capacity of the lung for carbon monoxide (DLCO), will be performed according to international guidelines. An overview of the study flow is shown in Fig. 1. After completion of the study, healthcare providers and research staff are asked to fill in a survey on their opinions and experiences with the home monitoring program in an online twelve item survey.

### I-FILE application

**Fig. 2:**
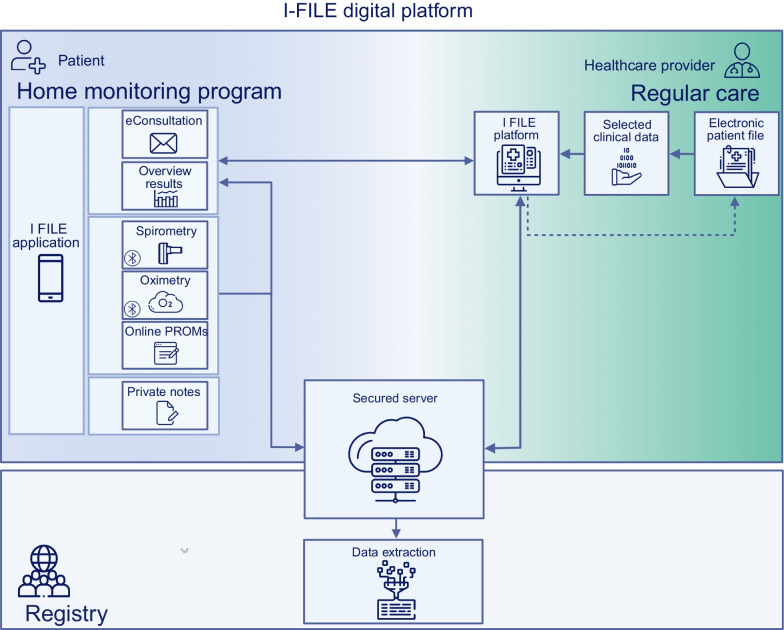
Digital platform of the I-FILE study

I-FILE is an online eHealth application (Curavista, Gezondheidsmeter, Geertruidenberg, the Netherlands) (www.i-file.app). This application has been successfully used in earlier (multi-center) home monitoring studies [23–24]. At time of inclusion, all patients will receive a password-protected personal account, and the I-FILE application will be installed on their smartphone or tablet in their native language. If patients do not own a smart device, they will be provided with a tablet computer for the duration of the study. The I-FILE application includes home spirometry, pulse oximetry, online completion of questionnaires and symptom scales, possibility for eConsultation with healthcare providers, an option for private notes for the patients, and a real-time overview of results over time (Fig. 2).

At first login, patients will be asked to tick a box to consent that their data can be stored in a database and used for research purposes; they can recall this consent at any time. All study results will be encrypted and directly sent to a secured and approved server (Gezondheidsmeter, the Netherlands), which has the highest European Certification for safety (NEN7510 and ISO27001), is CE-marked and complies with all safety regulations (i.e. General Data Protection Regulation).

Patients will be provided with an instruction manual on paper for the use of the I-FILE application. They will find additional information about the study on the I-FILE website, which includes animated instruction videos on the I-FILE study and the use of the spirometer. In addition, both patients and research staff will have access to a technical helpdesk in their native language.

Healthcare providers and research staff will receive a password for an online dashboard with an overview of all participating patients from their center. They will have real-time access to all patient data, except for the part with private notes. Some hospitals will have direct access to the I-FILE platform via the electronic patient file in their hospital. The data will be made available to all collaborating investigators and patient associations for different research questions and post-hoc data analyses.

Patients will receive automated reminders if measurements are not performed according to the study protocol. In case of repeated missing measurements, research staff will receive automated alerts with instructions to contact the patient. In addition, healthcare professionals are instructed to check the results of the included patient on a regular basis. Healthcare providers do not receive automated alerts in case of changes in pulmonary function, oxygen saturation or PROMs.

### Home spirometry and pulse oximetry

Home spirometry and oximetry measurements will be performed using a Bluetooth-enabled validated, CE-marked home spirometer (Spirobank Smart or Spirometer OXI MIR, Italy). Patients will be instructed by a trained member of the research team and will perform a minimum of three test blows on the home spirometer to ensure reliability of the home spirometry results. Home spirometry measurements will be considered reliable if the highest FVC value deviates less than ten percent from the in-hospital FVC value conducted at baseline, and if the difference between two consecutive measurements is less than 150 ml [28]. Patients are instructed to perform home spirometry approximately at the same time to reduce variability [29]. Oxygen saturation is measured in the subgroup of patients who use the Spirometer OXI, by placing their finger on the integrated sensor for 30 s. All measurements will be directly sent to the research team with a personal 5-digit code. Both patient and research team will have real-time access to the flow-volume loops, quality assessment of the measurements, and a trend line of the FVC and the FEV1 over time.

### Patient reported outcome measures

Patients will complete online PROMs (Table 2), consisting of HRQOL questionnaires and symptom scores, and can see a graphical overview of their main results.

**Table 2:** Patient reported outcome measures in the I-FILE study

	Description	Number of items and scoring	MCID	Reference
K-BILD	Self-completed health status questionnaire with 3 domains: breathlessness and activities, psychological, and chest symptoms	15 items Scores range from 0 to 100, with higher scores corresponding with better HRQOL	K-BILD total: 3.9 points	[30, 31]
L-PF	Questionnaire consists of 2 modules. The impacts module evaluates impact on daily activities. The symptom module evaluates shortness of breath, cough, and fatigue	44 items (Symptoms module: 23 items, Impacts module: 21 items) Scores range from 0 to 100, with higher scores indicating more impairment and lower HRQOL	L-PF Dyspnea: 6–7 points L-PF Cough: 4–5 points	[32, 33]
EQ-5D-5L	Questionnaire with 5 domains; mobility, self-care, usual activities, pain/discomfort, and anxiety/depression, combined with a VAS on health status with a scale from 0 to 100	6 items Higher score indicates better health status	EQ-5D-5L range: 0.0050–0.054 points EQ-VAS range: 0.5–5.0 points	[34, 35]
VAS	Symptom scores on cough, dyspnea, fatigue, and general wellbeing	1 item per symptom Scores range from 0 to 10, with higher scores on cough, dyspnea and fatigue reflecting more severe symptoms. Higher score on general wellbeing indicates better wellbeing	Not available	[19]
LCQ	Questionnaire on chronic cough	19 items The overall score ranges from 3 to 21, with a higher score indicating a better cough-related health status	1.3 points (IPF)	[36]
FAS	Self-administered questionnaire about fatigue	10 items The score ranges from 5 to 50 points, with a score of ≥ 22 points as cut-off for fatigue	4 points or 10% change (sarcoidosis)	[37, 38]
WPAI:GH	Questionnaire assessing impairment and productivity	6 items Outcomes are expressed as impairment percentages, with higher numbers indicating greater impairment and less productivity	Not available	[39]
GRoC	Global rating of change with regards to general well-being	1 item 15-point self-report scale (from − 7 to 7). Higher score indicates a better health condition compared to the previous study visit	5 points	[40]

*K-BILD* King’s brief Interstitial Lung Disease Health Status*, L-PF* the Living with Pulmonary Fibrosis*, EQ-5D-5L* The EuroQol five dimensions 5-level*, VAS* Visual Analogue Scale*, LCQ* Leicester Cough Questionnaire*, FAS* Fatigue Assessment Scale, *WPAI:GH* Work Productivity and Activity Impairment Questionnaire: General Health*, GRoC* The Global Rating of Change Scale

### Endpoints and statistical analysis

We will evaluate absolute changes in FVC (L and % predicted) measured with home spirometry, and oxygen saturation (%) measured with pulse oximetry, from baseline to 6, 12, and 24 months. These changes will be analyzed using a linear mixed model with disease and country as fixed effects. Repeated measurements within a patient will be corrected by random effects (intercepts or slopes, depending on the data). This model corrects for random missing data. Possible non-linear evolutions and correlations between subsequent observations will be accounted for through flexible modeling of the time variable and structured error terms in the variance–covariance matrix of the random effects. Absolute in-hospital FVC changes between baseline, 6, 12 and 24 months (L and %predicted), HRQOL, and symptom scores will also be assessed with linear mixed models. The correlation of home and hospital spirometry will be analyzed by calculating Pearson’s correlation coefficient, taking the individual mean FVC changes for each patient into account. Results will be used to optimize analytical methods to deal with outliers and missing data. Besides, a delta (Δ) change will be computed for HRQOL and symptom changes from baseline to 6, 12 and 24 months. Furthermore, we will use group based trajectory modeling and joint models to predict outcomes in individual patients (e.g. respiratory related and all cause hospitalizations, disease progression, and mortality) To measure time to decline in HRQOL, we will use the minimal clinically important difference (MCID) for all individual patient reported outcomes. Joint models will be used to assess time to reach the MCID. Adherence to the home monitoring program will be assessed by evaluating the completeness of data. The total number of measurements from each participant will be divided by the number of expected measurements, for pulmonary function as well as PROMs. The incidence rate of new oxygen prescription, non-elective hospitalization (respiratory and all cause), mortality (all cause and respiratory-related) will be summarized descriptively by diagnosis cohort. Disease progression will be defined according to the guideline criteria and its individual components, and will be compared with criteria used in different trials [16–18, 20]. We will also perform analyses with different cut-off values for FVC and DLCO. Multivariate logistic regression, ROC curve analysis and a multivariate Cox proportional hazards model will be used to identify possible predictive factors for disease progression and/or mortality. Differences between countries will be analyzed with ANOVA. If possible, HRQOL questionnaires will be validated for the different forms op PF by assessing internal consistency, discriminative ability, concurrent validity, and responsiveness. Patient and healthcare providers satisfaction with the home monitoring program will be summarized descriptively. An overview of the pre-specified endpoints is shown in Table 3.

**Table 3:** Prespecified endpoints of the I-FILE study

*Primary endpoint*
Absolute FVC change measured with home spirometry from baseline to 6, 12 and 24 months (in % and L)
*Secondary endpoints*
Absolute FVC change measured with in-hospital spirometry between baseline and 6, 12 and 24 months (in % and L) Absolute DLCO change measured with in-hospital spirometry between baseline and 6, 12 and 24 months (in % and L) Change in oxygen saturation from baseline to 6, 12 and 24 months (in %) Correlations between home and hospital spirometry Adherence to daily and weekly home spirometry HRQOL and symptom changes between baseline, 6, 12 and 24 months Time to change in HRQOL Correlations between pulmonary function tests and PROMs at all time points Internal consistency, discriminative ability, concurrent validity, and responsiveness of PROMs in individual diseases Hospitalizations (all cause and respiratory related) Mortality Percentage of patients with disease progression Identification of markers for disease progression or death Percentage of patients with new oxygen prescription Differences in outcomes between countries Differences in outcomes between subgroups of PF Healthcare provider and patient satisfaction and experience with the online application

*FVC* Forced vital capacity, *HRQOL* Health related quality of life, *PROMs* Patient reported outcome measures, *PF* Pulmonary fibrosis

## Discussion

The I-FILE study will be the first international patient registry for PF using online home monitoring. This study will help us gain better insights in disease course of different forms of PF through frequent collection of physiological parameters and PROMs and will yield important information about the feasibility of a patient registry using eHealth technology.

During the last decade, many patient registries have been established for PF, with most registries focusing on IPF. Registry data are especially of added value for rare diseases with a heterogeneous disease trajectory, to gain more real-life insights in disease behavior and response to treatment. Compared to randomized controlled trials, registries have less strict inclusion criteria, as they operate more aligned with daily clinical practice. Therefore, registries include patients with a broader range of disease severity, who are often older, have more pulmonary function impairment, and more comorbidities. Moreover, larger patient groups can be included and monitored for a longer time period. However, registry data also have drawbacks. Combining data from different registries is hampered by factors such as data ownership, diverse patient populations, and differences in study parameters and outcomes. Due to the study design and less strict in- and exclusion criteria, registries often encounter more missing and heterogeneous data compared to clinical trials. In addition, patients with more severe disease can possibly have a higher dropout rate, whereas patients with a relatively mild disease course may be lost to follow up if they are referred to local centers for follow up. Until now, most PF registries are retrospective and often included patients from a single country. The I-FILE study provides solutions to overcome these hurdles. The eHealth technology used in this study facilitates uniform and frequent data collection from different countries across Europe. Since patients are tracking their disease course at home, they can provide data for a long period of time independent of their treating center. Data entry in traditional registries is mostly conducted by healthcare providers or research staff and is often very time consuming. In the I-FILE study, we chose to place patients in the lead by giving them ownership of their own study data. Patients collect data and give authorization for using their data for scientific research. We hypothesize that, compared to existing registry processes, patient-led data registration is less laborious for healthcare providers and research staff, and provides a more practicable way of frequent data logging at minimal burden for patients. The experience and satisfaction of all stake holders will be evaluated.

Results of this study will provide insights into disease behavior across different forms of PF. Because of the sample size and duration, we expect to be able to evaluate relatively large subgroups of individual diseases and rare events, such as acute exacerbations. Frequent home spirometry measurements may facilitate early detection of acute exacerbations and disease progression. This is particularly relevant in this patient population, since there is a paucity of prognostic and predictive biomarkers. Data from this study can shed light on whether the use of home spirometry can optimize timing of treatment initiation for individual patients. The I-FILE study will also reveal potential differences in clinical management and outcomes of patients with PF across different European countries.

Earlier studies have elucidated the potential benefits of online home monitoring for patients with PF, both for regular care and research purposes. A randomized controlled trial in IPF found that an online home monitoring program, including home spirometry and online completion of patient-reported outcome measures, tended to improve psychological wellbeing compared to standard care, and was highly appreciated by patients. In addition, online home monitoring allowed for early treatment adjustments in individual patients in case of pulmonary function decline or side-effects of treatment [22]. Results of the I-FILE will build on these insights and show whether home monitoring of patients with potentially progressive disease remains viable and beneficial on the long-term. We postulate that real-time access to pulmonary function, symptoms, and HRQOL data can enhance patient engagement and self-management, as patients gain more insights in their own disease course. The I-FILE study will also reveal whether disease course has influence on the adherence to home monitoring. For research purposes, frequent online data collection using home monitoring can facilitate early identification of patients with a progressive phenotype for inclusion in clinical trials, as well as possibilities for real-time safety monitoring, and broader access to trials [21].

Technical issues are frequently mentioned as expected hurdles for further implementation of eHealth technology in clinical practice. We aim to overcome this hurdle with a technical helpdesk in the native language, accessible for patients, healthcare providers, and research staff. Furthermore, the home monitoring program used in the I-FILE study has been co-developed together with patients and extensively evaluated in earlier studies [23–24, 25]. We will host an annual patient meeting to actively incorporate patient’s experience with the platform, their perspectives on study and will also encourage input on research questions from all stakeholders. We hope that in this way the traditional model of registries where patients “deliver” data and researcher analyze, will gradually shift to a more collaborative effort. Insights gained in this study can be used to further optimize eHealth applications and collaborative registries for patients with PF in the future.

In this study, we will also collect longitudinal data about HRQOL, symptoms, and disease burden. Although PROMs are increasingly used in clinical trials, many have not been validated in different forms of PF or in real-life settings, which we aim to do in the current study. Besides clinical trials, PROMs can also be used in daily practice, although it can be time consuming or difficult for patients to complete lengthy PROMs on a regular basis. Previous research indicated that the use of online visual analogue scales in patients with IPF is feasible and reliable, and the visual analogue scale is an easy tool to assess symptoms over time [41]. Here, we aim to validate and extend these results in a larger and more diverse patient population over a prolonged period of time.

Although lessons were learned from previous eHealth studies conducted in patients with PF, we still anticipate challenges during the setup and throughout the execution of the study. Since the I-FILE will be conducted in different countries, we anticipate the initiation of the study to be complicated by differences in legislation between countries with regard to home monitoring. During the conduct of the study, one of the anticipated issues could be a lack of internet access or technological skills in a subgroup of patients. Therefore, one of our aims is to evaluate feasibility of this international patient-led registry and gain more insights in differences between countries.

In conclusion, the I-FILE registry study is the ultimate collaboration between researchers, healthcare providers and patients. This study will yield valuable information about the clinical course of patients with PF and potential differences in disease management across Europe. Simultaneously, the I-FILE study will provide broad insights on the use and feasibility of home-based data collection. This international patient-led registry can facilitate trans-border collaboration to further optimize care and research for patients with PF.

## Supplementary Information

**Additional file 1: Table S1. **Data collection I-FILE study.

## Data Availability

The full protocol is available from the corresponding author on reasonable request.

## Abbreviations

PF: Pulmonary fibrosis
PROMs: Patient reported outcome measures
K-BILD: The King’s brief Interstitial Lung Disease Health Status
EQ5D-5L: The EuroQol five dimensions five-level
VAS: Visual analogue scale
LCQ: Leicester cough questionnaire
FAS: Fatigue assessment scale
WPAI: Work productivity and activity impairment questionnaire
GRoC: Global rating of change scale
L-PF: Living with pulmonary fibrosis
HRQOL: Health-related quality of life
IPF: Idiopathic pulmonary fibrosis
PPF: Non-IPF progressive pulmonary fibrosis
FVC: Forced vital capacity
MDD: Multidisciplinary-discussion
FEV1: Forced expiratory volume in 1s
DLCO: Diffusion capacity of the lung for carbon monoxide
