# Supplementary material for: Study protocol of an international patient-led registry in patients with pulmonary fibrosis using online home monitoring: I-FILE
Source: BMC Pulm Med. 2023 Feb 2;23:51. doi: 10.1186/s12890-023-02336-4 (PMC9893651; doi:10.1186/s12890-023-02336-4)
Supplement: Supplementary file 1 — Additional file 1: Table S1. Data collection I-FILE study. [file 12890_2023_2336_MOESM1_ESM.docx]

Supplementary table 1. Data collection I-FILE study

| **Data collection at baseline** |
| --- |
| **Demography**  Age  Gender  Height  Weight  Smoking status  Place of residence  Diagnosis  IPF  iNSIP  HP  CTD-ILD  SSc-ILD  RA-ILD  Sjogren  Polymyositis/Dermatomyositis  UCTD/Mixed MCTD  Other  Medication induced ILD  RB - ILD  DIP  Pneumoconiosis  Asbestosis  Silicosis  Other  PPFE  Unclassifiable ILD  Sarcoidosis (scadding stage IV)  Other  Date of diagnosis  Comorbidity  Coronary heart disease  Cerebrovascular disease  Atrial fibrillation  Deep venous thrombosis  Pulmonary embolism  Systemic Hypertension  Arterial hypertension  Valvular heart disease  Gastro esophageal reflux  Diabetes mellitus  Lung cancer  Obstructive sleep apnea  Depression/depressive disorder  Other  Environmental exposure  Occupational  Hobby  Fibrosis in family |
| **Imaging**  CT Scan pattern  If diagnosis is IPF  Typical UIP  Probable UIP  Indeterminate  Most consistent with non-IPF  If diagnosis is Non IPF  Predominant distribution  Upper lobe  Basal  Diffuse  Central  Peripheral  Predominant features  Air trapping  Ground – glass opacification  Honeycombing  Traction bronchiectasis/bronchiolectasis  Fine reticulation  Consolidation  Pleural involvement |
| **Pathology**  Technique  Surgical lung biopsy  Cryobiopsy  Pattern lung biopsy |
| **Serologic evaluation**  Rheumatoid factor  Anti-cyclic citrullinated peptide  Anti-nuclear antibodies  SS-A  SS-B  Scleroderma blot  Myositis blot  Others  Genetic testing  Detected mutations |
| **Pulmonary function test – in hospital**  TLC (L and %predicted)  FVC (L and %predicted)  FEV1 (L and %predicted)  FEV1/(F)VC (% and %predicted)  DLCO (mmol/(min*kPa) and %predicted)  Blood oxygen saturation (%)  Supplemental oxygen: if yes … (L/min)  6 MWT  Distance (m)  Saturation (before) (%)  Saturation (lowest) (%) |
| **Medication for ILD***  Medication  Pirfenidone  Nintedanib  Mycophenolate mofetil  Cyclofosfamide  Corticosteroids  Azathioprine  Rituximab  Methotrexate  Maintenance antibiotics  Other  Start date  Dosage |
| **Data collection at 6 months, 12 months, 18 months and 24 months** |
| **Hospital admission**  All cause  Respiratory related  **Medication changes for ILD***  Medication  Pirfenidone  Nintedanib  Mycophenolate mofetil  Cyclofosfamide  Corticosteroids  Azathioprine  Rituximab  Methotrexate  Maintenance antibiotics  Other  Start date  Dosage  **Side effects related to ILD medication**  Gastrointestinal  Nausea  Vomiting  Diarrhea  Flatulence  Abdominal discomfort  Reflux  Decreased appetite / lack of appetite  Abnormal liver enzymes  Other  Skin  Rash  Sunburn  Other skin problems such as dry skin or itching  Other  Headache  Insomnia  Fatigue  Dizziness  Renal impairment  Infections  Upper respiratory tract  Lower respiratory tract  Urinary tract  Weight change  Decrease  Increase  Other |
| **Pulmonary function test – in hospital**  TLC (L and %predicted)  FVC (L and %predicted)  FEV1 (L and %predicted)  FEV1/(F)VC (% and %predicted)  DLCO (mmol/(min*kPa) and %predicted)  Blood oxygen saturation (%)  Supplemental oxygen: if yes … (L/min)  6 MWT  Distance (m)  Saturation (before) (%)  Saturation (lowest) (%) |

*Data on duration, dose reduction, switches and discontinuations will be collected.
